# 2-Amino-5-chloro­pyridinium (*Z*)-3-carb­oxy­prop-2-enoate 0.25-hydrate

**DOI:** 10.1107/S1600536810033507

**Published:** 2010-08-28

**Authors:** Madhukar Hemamalini, Hoong-Kun Fun

**Affiliations:** a X-ray Crystallography Unit, School of Physics, Universiti Sains Malaysia, 11800 USM, Penang, Malaysia

## Abstract

In the title hydrated salt, C_5_H_6_ClN_2_^+^·C_4_H_3_O_4_^−^·0.25H_2_O, the water O atom lies on a twofold axis with 0.25 occupancy. The 2-amino-5-chloro­pyridinium cation is almost planar, with a maximum deviation of 0.015 (3) Å. In the hydrogen malate anion, an intra­molecular O—H⋯O hydrogen bond generates an *S*(7) ring and results in a folded conformation. In the crystal, the protonated N atom and the 2-amino group of the cation are hydrogen bonded to the carboxyl­ate O atoms of the anion *via* a pair of N—H⋯O hydrogen bonds, forming an *R*_2_^2^(8) ring motif. The ion pairs are further connected *via* O—H⋯O, N—H⋯O and C—H⋯O hydrogen bonds, forming layers parallel to the *ab* plane which stack down the *c* axis.

## Related literature

For hydrogen bonds in supra­molecular assemblies, see: Aakeröy & Seddon (1993 ▶); Fredericks & Hamilton (1996 ▶). For related structures of maleate salts, see: Rajagopal *et al.* (2001*a* ▶,*b* ▶, 2002 ▶); Alagar *et al.* (2001 ▶). For hydrogen-bond motifs, see: Bernstein *et al.* (1995 ▶). For the stability of the temperature controller used in the data collection, see: Cosier & Glazer (1986 ▶).
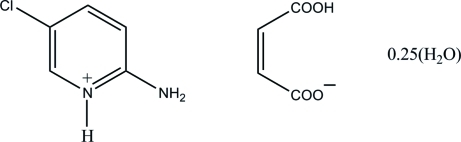


## Experimental

### 

#### Crystal data

C_5_H_6_ClN_2_^+^·C_4_H_3_O_4_^−^·0.25H_2_O
*M* *_r_* = 249.14
Orthorhombic, 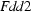

*a* = 23.899 (4) Å
*b* = 48.298 (8) Å
*c* = 3.7314 (7) Å
*V* = 4307.1 (13) Å^3^
*Z* = 16
Mo *K*α radiation
μ = 0.36 mm^−1^
*T* = 100 K
0.90 × 0.09 × 0.07 mm

#### Data collection

Bruker APEXII DUO CCD diffractometer
Absorption correction: multi-scan (*SADABS*; Bruker, 2009 ▶) *T*_min_ = 0.739, *T*_max_ = 0.976
8113 measured reflections
3485 independent reflections
2896 reflections with *I* > 2σ(*I*)
*R*_int_ = 0.037

#### Refinement

*R*[*F* ^2^ > 2σ(*F* ^2^)] = 0.048
*wR*(*F* ^2^) = 0.109
*S* = 1.08
3485 reflections
151 parameters
1 restraint
H-atom parameters constrained
Δρ_max_ = 0.28 e Å^−3^
Δρ_min_ = −0.28 e Å^−3^
Absolute structure: Flack (1983 ▶), 1354 Fridel pairs
Flack parameter: 0.03 (8)

### 

Data collection: *APEX2* (Bruker, 2009 ▶); cell refinement: *SAINT* (Bruker, 2009 ▶); data reduction: *SAINT*; program(s) used to solve structure: *SHELXTL* (Sheldrick, 2008 ▶); program(s) used to refine structure: *SHELXTL*; molecular graphics: *SHELXTL*; software used to prepare material for publication: *SHELXTL* and *PLATON* (Spek, 2009 ▶).

## Supplementary Material

Crystal structure: contains datablocks global, I. DOI: 10.1107/S1600536810033507/hb5596sup1.cif

Structure factors: contains datablocks I. DOI: 10.1107/S1600536810033507/hb5596Isup2.hkl

Additional supplementary materials:  crystallographic information; 3D view; checkCIF report

## Figures and Tables

**Table 1:** Hydrogen-bond geometry (Å, °)

*D*—H⋯*A*	*D*—H	H⋯*A*	*D*⋯*A*	*D*—H⋯*A*
N1—H1*N*1⋯O1	0.82	1.91	2.714 (3)	166
N2—H1*N*2⋯O2	0.86	2.04	2.870 (3)	161
N2—H2*N*2⋯O4	0.86	2.05	2.902 (3)	169
O3—H1*O*3⋯O2	0.95	1.53	2.447 (2)	162
O1*W*—H1*W*1⋯O1	0.82	2.00	2.718 (4)	146
C3—H3*A*⋯O4^i^	0.93	2.50	3.388 (3)	160
C4—H4*A*⋯O3	0.93	2.42	3.263 (3)	151

Symmetry code: (i) 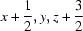.

**Fig. 1.**
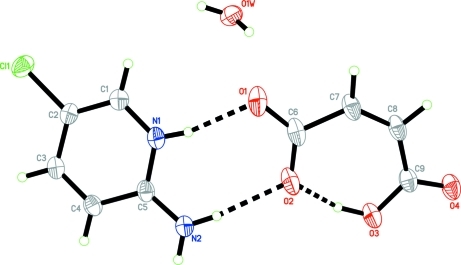
The asymmetric unit of the title compound. Displacement ellipsoids are drawn at the 50% probability level. Dashed lines indicate hydrogen bonds.

**Fig. 2.**
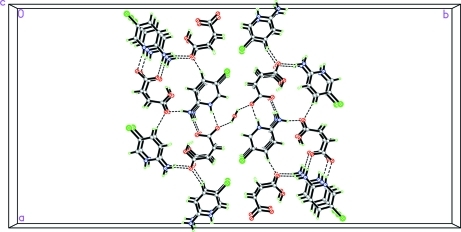
The crystal packing of (I), showing hydrogen-bonded (dashed lines) 2D networks parallel to to the ab-plane. H atoms not involved in the intermolecular interactions have been omitted for clarity.

## supplementary crystallographic information

### Comment

There is growing interest in the construction of supramolecular assemblies with hydrogen bonds as the building blocks (Aakeröy & Seddon, 1993; Fredericks & Hamilton, 1996). The maleic acid anion can exist in the fully deprotonated form or as hydrogen maleate, with one of the carboxylic acid groups protonated.  The crystal structures of glycinium maleate (Rajagopal *et al.*, 2001*a*), β-alaninium maleate (Rajagopal *et al.*, 2001*b*), sarcosinium maleate (Rajagopal *et al.*, 2002) and L-alaninium maleate (Alagar *et al.*, 2001) have been reported in the literature.  The present study reports the crystal structure of 2-amino-5-chloropyridinium hydrogen maleate 0.25-hydrate, (I), a complex of 2-amino-5-chloropyridinium with maleic acid.

The asymmetric unit (Fig. 1), contains a 2-amino-5-chloropyridinium cation, a hydrogen malate anion and a water molecule with occupany 0.25 (the O atom of the water molecule lies on a twofold axis). The 2-amino-5-chloropyridinium cation is essentially planar, with a maximum deviation of 0.015 (3) Å for atom C1. In the 2-amino-5-chloropyridinium cation, a wide angle [C1—N1—C5 = 123.39 (18)°] is subtended at the protonated N1 atom.  The dihedral angle between the pyridine ring and the mean plane formed by the hydrogen maleate anion is 22.39 (10)°.

In the crystal packing, the protonated N1 atom and the 2-amino group (N2) are hydrogen-bonded to the carboxylate oxygen atoms (O1 and O2) via a pair of intermolecular N1—H1N1···O1 and N2—H1N2···O2 hydrogen bonds, forming a ring motif *R*_2_^2^(8) (Bernstein *et al.*, 1995). The ion pairs are further connected via N2—H2N2···O4, O1W—H1W1···O1, C3—H3A···O4 and C4—H4A···O3 (Table 1) hydrogen bonds, forming two-dimensional networks parallel to the *ab* plane (Fig. 2) which stacked down the *c*-axis. In the hydrogen malate anion, an intramolecular O3—H1O3···O2 hydrogen bond generates an *S*(7) ring and results in a folded conformation.

### Experimental

A hot methanol solution (20 ml) of 2-amino-5-chloropyridine (64 mg, Aldrich) and maleic acid (58 mg, Merck) were mixed and warmed over a heating magnetic stirrer hotplate for a few minutes. The resulting solution was allowed to cool slowly at room temperature and colourless needles of (I) appeared after a few days.

### Refinement

All hydrogen atoms were positioned geometrically [C—H = 0.93 Å, N—H = 0.8196–0.86 Å and O—H = 0.8190–0.9462 Å] and were refined using a riding model, with *U*_iso_(H) = 1.2 *U*_eq_(C, N) or 1.5 *U*_eq_(O). 1354 Friedel pairs were used to determine the absolute structure.

### Crystal data

**Table:**

C_5_H_6_ClN_2_^+^·C_4_H_3_O_4_^−^·0.25H_2_O	*F*(000) = 2056
*M**_r_* = 249.14	*D*_x_ = 1.537 Mg m^−^^3^
Orthorhombic, *F**d**d*2	Mo *K*α radiation, λ = 0.71073 Å
Hall symbol: F 2 -2d	Cell parameters from 2106 reflections
*a* = 23.899 (4) Å	θ = 3.1–30.2°
*b* = 48.298 (8) Å	µ = 0.36 mm^−^^1^
*c* = 3.7314 (7) Å	*T* = 100 K
*V* = 4307.1 (13) Å^3^	Needle, colourless
*Z* = 16	0.90 × 0.09 × 0.07 mm

### Data collection

**Table:**

Bruker APEXII DUO CCD diffractometer	3485 independent reflections
Radiation source: fine-focus sealed tube	2896 reflections with *I* > 2σ(*I*)
graphite	*R*_int_ = 0.037
φ and ω scans	θ_max_ = 32.1°, θ_min_ = 3.1°
Absorption correction: multi-scan (*SADABS*; Bruker, 2009)	*h* = −35→19
*T*_min_ = 0.739, *T*_max_ = 0.976	*k* = −72→72
8113 measured reflections	*l* = −5→5

### Refinement

**Table:**

Refinement on *F*^2^	Secondary atom site location: difference Fourier map
Least-squares matrix: full	Hydrogen site location: inferred from neighbouring sites
*R*[*F*^2^ > 2σ(*F*^2^)] = 0.048	H-atom parameters constrained
*wR*(*F*^2^) = 0.109	*w* = 1/[σ^2^(*F*_o_^2^) + (0.0339*P*)^2^ + 6.9914*P*] where *P* = (*F*_o_^2^ + 2*F*_c_^2^)/3
*S* = 1.08	(Δ/σ)_max_ < 0.001
3485 reflections	Δρ_max_ = 0.28 e Å^−^^3^
151 parameters	Δρ_min_ = −0.28 e Å^−^^3^
1 restraint	Absolute structure: Flack (1983), 1354 Fridel pairs
Primary atom site location: structure-invariant direct methods	Flack parameter: 0.03 (8)

### Special details

**Table:**

Experimental. The crystal was placed in the cold stream of an Oxford Cryosystems Cobra open-flow nitrogen cryostat (Cosier & Glazer, 1986) operating at 100.0 (1) K.
Geometry. All s.u.'s (except the s.u. in the dihedral angle between two l.s. planes) are estimated using the full covariance matrix. The cell s.u.'s are taken into account individually in the estimation of s.u.'s in distances, angles and torsion angles; correlations between s.u.'s in cell parameters are only used when they are defined by crystal symmetry. An approximate (isotropic) treatment of cell s.u.'s is used for estimating s.u.'s involving l.s. planes.
Refinement. Refinement of F^2^ against ALL reflections. The weighted R-factor wR and goodness of fit S are based on F^2^, conventional R-factors R are based on F, with F set to zero for negative F^2^. The threshold expression of F^2^ > 2σ(F^2^) is used only for calculating R-factors(gt) etc. and is not relevant to the choice of reflections for refinement. R-factors based on F^2^ are statistically about twice as large as those based on F, and R- factors based on ALL data will be even larger.

### Fractional atomic coordinates and isotropic or equivalent isotropic displacement parameters (Å^2^)

**Table:**

	*x*	*y*	*z*	*U*_iso_*/*U*_eq_	Occ. (<1)
Cl1	0.69696 (2)	0.016473 (11)	0.8123 (2)	0.03527 (16)	
N1	0.55035 (7)	0.05354 (4)	0.7539 (6)	0.0249 (4)	
H1N1	0.5183	0.0512	0.6816	0.030*	
N2	0.51706 (8)	0.09744 (4)	0.8771 (6)	0.0314 (5)	
H1N2	0.4843	0.0922	0.8093	0.038*	
H2N2	0.5223	0.1141	0.9500	0.038*	
C1	0.59109 (8)	0.03419 (4)	0.7366 (7)	0.0247 (4)	
H1A	0.5830	0.0164	0.6549	0.030*	
C2	0.64399 (8)	0.04084 (4)	0.8395 (7)	0.0238 (4)	
C3	0.65571 (9)	0.06771 (4)	0.9713 (6)	0.0252 (4)	
H3A	0.6917	0.0723	1.0464	0.030*	
C4	0.61411 (9)	0.08674 (4)	0.9871 (6)	0.0251 (4)	
H4A	0.6215	0.1044	1.0751	0.030*	
C5	0.55917 (8)	0.07983 (4)	0.8695 (6)	0.0243 (4)	
O1	0.44806 (7)	0.03654 (4)	0.5270 (6)	0.0387 (4)	
O2	0.41927 (7)	0.08014 (4)	0.4907 (5)	0.0368 (4)	
O3	0.34524 (7)	0.10600 (3)	0.1851 (5)	0.0331 (4)	
H1O3	0.3779	0.0991	0.2975	0.050*	
O4	0.26906 (7)	0.09571 (3)	−0.1213 (6)	0.0354 (4)	
C6	0.41315 (9)	0.05435 (5)	0.4340 (7)	0.0314 (5)	
C7	0.36132 (9)	0.04403 (5)	0.2533 (7)	0.0309 (5)	
H7A	0.3578	0.0249	0.2461	0.037*	
C8	0.31932 (9)	0.05795 (5)	0.1004 (7)	0.0289 (5)	
H8A	0.2910	0.0468	0.0086	0.035*	
C9	0.30996 (9)	0.08816 (5)	0.0515 (7)	0.0280 (5)	
O1W	0.5000	0.0000	0.0840 (17)	0.0348 (11)	0.50
H1W1	0.4736	0.0067	0.1941	0.052*	0.50

### Atomic displacement parameters (Å^2^)

**Table:**

	*U*^11^	*U*^22^	*U*^33^	*U*^12^	*U*^13^	*U*^23^
Cl1	0.0224 (2)	0.0272 (2)	0.0563 (4)	0.00083 (19)	0.0022 (3)	0.0053 (3)
N1	0.0196 (7)	0.0327 (8)	0.0223 (10)	−0.0017 (6)	−0.0009 (8)	0.0005 (7)
N2	0.0242 (8)	0.0334 (9)	0.0365 (13)	0.0029 (7)	−0.0077 (9)	−0.0069 (9)
C1	0.0244 (9)	0.0272 (9)	0.0225 (11)	−0.0014 (7)	0.0012 (9)	0.0035 (8)
C2	0.0208 (8)	0.0274 (8)	0.0232 (11)	0.0006 (7)	0.0015 (9)	0.0062 (9)
C3	0.0212 (9)	0.0319 (10)	0.0224 (11)	−0.0026 (7)	−0.0018 (8)	0.0038 (9)
C4	0.0253 (9)	0.0296 (9)	0.0206 (11)	−0.0029 (7)	−0.0013 (8)	0.0007 (9)
C5	0.0232 (9)	0.0323 (9)	0.0175 (11)	−0.0003 (7)	−0.0022 (9)	0.0014 (9)
O1	0.0255 (7)	0.0530 (9)	0.0376 (11)	−0.0024 (7)	−0.0090 (8)	0.0088 (10)
O2	0.0282 (8)	0.0445 (9)	0.0377 (11)	−0.0109 (7)	−0.0125 (8)	0.0055 (8)
O3	0.0286 (8)	0.0378 (8)	0.0329 (10)	−0.0097 (6)	−0.0083 (7)	0.0059 (7)
O4	0.0291 (8)	0.0396 (8)	0.0376 (11)	−0.0043 (7)	−0.0123 (8)	0.0101 (8)
C6	0.0231 (10)	0.0481 (13)	0.0231 (12)	−0.0091 (9)	−0.0039 (9)	0.0069 (10)
C7	0.0266 (10)	0.0370 (11)	0.0293 (13)	−0.0111 (8)	−0.0072 (10)	0.0117 (10)
C8	0.0238 (9)	0.0372 (10)	0.0256 (12)	−0.0113 (8)	−0.0061 (9)	0.0117 (10)
C9	0.0238 (9)	0.0360 (10)	0.0241 (12)	−0.0067 (8)	−0.0006 (9)	0.0062 (10)
O1W	0.033 (2)	0.0282 (19)	0.043 (3)	0.0037 (17)	0.000	0.000

### Geometric parameters (Å, °)

**Table:**

Cl1—C2	1.731 (2)	C4—H4A	0.9300
N1—C1	1.351 (3)	O1—C6	1.248 (3)
N1—C5	1.357 (3)	O2—C6	1.272 (3)
N1—H1N1	0.8196	O3—C9	1.304 (3)
N2—C5	1.318 (3)	O3—H1O3	0.9462
N2—H1N2	0.8600	O4—C9	1.226 (3)
N2—H2N2	0.8600	C6—C7	1.496 (3)
C1—C2	1.360 (3)	C7—C8	1.336 (3)
C1—H1A	0.9300	C7—H7A	0.9300
C2—C3	1.416 (3)	C8—C9	1.488 (3)
C3—C4	1.355 (3)	C8—H8A	0.9300
C3—H3A	0.9300	O1W—H1W1	0.8190
C4—C5	1.424 (3)		
			
C1—N1—C5	123.39 (18)	C5—C4—H4A	119.9
C1—N1—H1N1	124.1	N2—C5—N1	119.47 (19)
C5—N1—H1N1	112.3	N2—C5—C4	123.1 (2)
C5—N2—H1N2	120.0	N1—C5—C4	117.41 (18)
C5—N2—H2N2	120.0	C9—O3—H1O3	117.9
H1N2—N2—H2N2	120.0	O1—C6—O2	123.5 (2)
N1—C1—C2	119.54 (19)	O1—C6—C7	116.7 (2)
N1—C1—H1A	120.2	O2—C6—C7	119.8 (2)
C2—C1—H1A	120.2	C8—C7—C6	130.3 (2)
C1—C2—C3	119.90 (19)	C8—C7—H7A	114.8
C1—C2—Cl1	120.18 (17)	C6—C7—H7A	114.8
C3—C2—Cl1	119.92 (15)	C7—C8—C9	131.2 (2)
C4—C3—C2	119.45 (19)	C7—C8—H8A	114.4
C4—C3—H3A	120.3	C9—C8—H8A	114.4
C2—C3—H3A	120.3	O4—C9—O3	121.3 (2)
C3—C4—C5	120.3 (2)	O4—C9—C8	118.4 (2)
C3—C4—H4A	119.9	O3—C9—C8	120.2 (2)
			
C5—N1—C1—C2	0.2 (3)	C3—C4—C5—N2	−179.8 (2)
N1—C1—C2—C3	1.4 (3)	C3—C4—C5—N1	2.0 (3)
N1—C1—C2—Cl1	−178.96 (18)	O1—C6—C7—C8	−174.0 (3)
C1—C2—C3—C4	−1.2 (4)	O2—C6—C7—C8	7.2 (4)
Cl1—C2—C3—C4	179.14 (19)	C6—C7—C8—C9	1.2 (5)
C2—C3—C4—C5	−0.5 (4)	C7—C8—C9—O4	175.2 (3)
C1—N1—C5—N2	179.8 (2)	C7—C8—C9—O3	−3.6 (4)
C1—N1—C5—C4	−1.9 (3)		

### Hydrogen-bond geometry (Å, °)

**Table:**

*D*—H···*A*	*D*—H	H···*A*	*D*···*A*	*D*—H···*A*
N1—H1N1···O1	0.82	1.91	2.714 (3)	166
N2—H1N2···O2	0.86	2.04	2.870 (3)	161
N2—H2N2···O4^i^	0.86	2.05	2.902 (3)	169
O3—H1O3···O2	0.95	1.53	2.447 (2)	162
O1W—H1W1···O1	0.82	2.00	2.718 (4)	146
C3—H3A···O4^ii^	0.93	2.50	3.388 (3)	160
C4—H4A···O3^i^	0.93	2.42	3.263 (3)	151

Symmetry codes: (i) ; (ii) *x*+1/2, *y*, *z*+3/2.
